# Economic evaluation of peritoneal dialysis and hemodialysis in Thai population with End-stage Kidney Disease

**DOI:** 10.1186/s12913-022-08827-0

**Published:** 2022-11-21

**Authors:** Montira Assanatham, Oraluck Pattanaprateep, Anan Chuasuwan, Kriengsak Vareesangthip, Ouppatham Supasyndh, Adisorn Lumpaopong, Paweena Susantitaphong, Chutatip Limkunakul, Wanchana Ponthongmak, Kamolpat Chaiyakittisopon, Ammarin Thakkinstian, Atiporn Ingsathit

**Affiliations:** 1 Mahidol University Health Technology Assessment (MUHTA) Graduate Program, Mahidol University, 10400 Bangkok, Thailand; 2 Division of Nephrology, Department of Medicine, Faculty of Medicine, Ramathibodi Hospital, Mahidol University, Bangkok, Thailand; 3 Department of Clinical Epidemiology and Biostatistics, Faculty of Medicine, Ramathibodi Hospital, Mahidol University, 270 Rama VI Road., Ratchathewi, Bangkok, Thailand; 4 Nephrology Division, Department of Medicine, Bhumibol Adulyadej Hospital, 10220 Bangkok, Thailand; 5 Renal Division, Department of Medicine, Faculty of Medicine Siriraj Hospital, Mahidol University, Bangkok, Thailand; 6 Division of Nephrology, Department of Medicine, Phramongkutklao Hospital and College of Medicine, Bangkok, Thailand; 7 Pediatric Nephrology Division, Department of Pediatrics, Phramongkutklao Hospital, Bangkok, Thailand; 8 Division of Nephrology, Department of Medicine, King Chulalongkorn Memorial Hospital, Chulalongkorn University, Bangkok, Thailand; 9 Division of Nephrology, Panyananthaphikkhu Chonprathan Medical Center, Srinakharinwirot University, Bangkok, Thailand; 10 Department of Community Pharmacy, Faculty of Pharmacy, Silpakorn University, Nakorn Pathom, Thailand

**Keywords:** Hemodialysis, Peritoneal dialysis, Economic evaluation, End-stage kidney disease

## Abstract

**Background:**

This study aimed to conduct a cost-utility analysis of the “Peritoneal Dialysis (PD)-First” policy in 2008 under a universal health coverage scheme and hemodialysis (HD) in Thai patients with End-stage Kidney Disease (ESKD) using updated real-practice data.

**Methods:**

Markov model was used to evaluate the cost-utility of two modalities, stratified into five age groups based on the first modality taken at 20, 30, 40, 50, and 60 years old from government and societal perspectives. Input parameters related to clinical aspects and cost were obtained from 15 hospitals throughout Thailand and Thai Renal Replacement Therapy databases. Both costs and outcomes were discounted at 3%, adjusted to 2021, and converted to USD (1 USD = 33.57 Thai Baht). One-way analysis and probabilistic sensitivity analysis were performed to assess the uncertainty surrounding model parameters.

**Results:**

From the government perspective, compared to PD-first policy, the incremental cost-effectiveness ratio (ICER) was between 19,434 and 23,796 USD per QALY. Conversely, from a societal perspective, the ICER was between 31,913 and 39,912 USD per QALY. Both are higher than the willingness to pay threshold of 4,766 USD per QALY.

**Conclusion:**

By applying the updated real-practice data, PD-first policy still remains more cost-effective than HD-first policy at the current willingness to pay. However, HD gained more quality-adjusted life years than PD. This information will assist clinicians and policymakers in determining the future direction of dialysis modality selection and kidney replacement therapy reimbursement policies for ESKD patients.

**Supplementary Information:**

The online version contains supplementary material available at 10.1186/s12913-022-08827-0.

## Introduction

End-stage Kidney Disease (ESKD) is one of the most important global public health challenges due to its increasing prevalence, high mortality rate, and high cost of treatment [1, 2]. Consequently, kidney replacement therapy, including peritoneal dialysis (PD), haemodialysis (HD), or kidney transplantation (KT), is necessary to maintain the lives of ESKD patients. Albeit, KT is ranked as the most successful treatment to prolong survival, improve quality of life, and the most cost-effective treatment option for ESKD patients [3–5]. However, the accessibility of KT is limited due to organ donor shortages [6]. In overall, kidney replacement therapy has accounted for healthcare expenditures of about 2–4% in some countries [7] and 3% in Thailand [8, 9].

Thailand has started the implementation of a “PD-first” policy with full reimbursement as a part of the universal health coverage system since 2008. Concurrently, the policy is based on a prior economic evaluation study in 2007 [10], which compared continuous ambulatory peritoneal dialysis (CAPD) or HD with palliative care. The study showed that providing PD as initial treatment was preferable to HD due to the assumption of PD and HD survival rates were similar. However, previous studies showed conflict in overall survival, so PD might prolong survival [11–14], similarly [15–17], or shorten survival to HD [14, 18, 19]. A recent finding from Thailand also showed that the PD-first policy had worse survival outcomes than HD [20]. Since there has been an improvement in the quality of care over time, this may result in prolonged survival and the quality of life of PD patients. In addition, purchasing power from central price negotiation could dramatically decrease the cost of PD relative to the period preceding policy implementation [21]; so these together may impact the cost-effectiveness analysis outcomes A cost-effectiveness analyses of HD and PD has not been updated since PD-first policy was implemented. Therefore, we aimed to conduct a cost-utility analysis (CUA) of PD and HD in Thai patients with ESKD using up-to-date, real-practice data from PD and HD patients.

## Methods

### Study design

The study was a CUA applying a Markov model and decision tree analysis (TreeAge Pro® 2021) [22]. Lifetime horizon was applied with societal and government perspectives. The study’s protocol was approved by the IRB of the Ministry of Public Health (Ref. no. 28/2561), and local ethic committees if required. Our study consisted of 2 phases: First, we invited 39 hospitals, but only 15 hospitals agreed to participate and provided clinical data from 2010 to 2018 for estimating transition probability, see Additional file 1: Appendix A. Second, we conducted a cross-sectional survey based on 11 hospitals that were selected representing each region of Thailand in order to collect direct non-medical costs, indirect costs, and utility. A stratified-cluster random sampling was applied to select the patients from each selected hospital for interviews and collect direct non-medical and indirect costs and utility from January to May 2020. As a result, a total of 660 patients (PD = 293, HD = 367) were willing to participate and included in the analysis.

### Interventions of interest

The interventions of interest were PD and HD. PD is a procedure using peritoneal lining that acts as a membrane to allow excess fluids and waste products to diffuse from the bloodstream into the dialysis fluid, which typically can be done at home. However, patients who prefer PD should be able to understand how to set up the equipment and use their hands to connect and disconnect small tubes to prevent infections. HD is a procedure that pumps a patient’s blood out from the body through a dialysis machine to remove waste products and excess fluids and then return to the body, needing surgically created vascular access. HD is usually performed at a dialysis centre in Thailand, two or three times a week, and takes about three to five hours per session. HD is more beneficial than PD in terms of efficient clearance and short duration time for treatment, but require transport expenses.

### Model structure

**Fig. 1:**
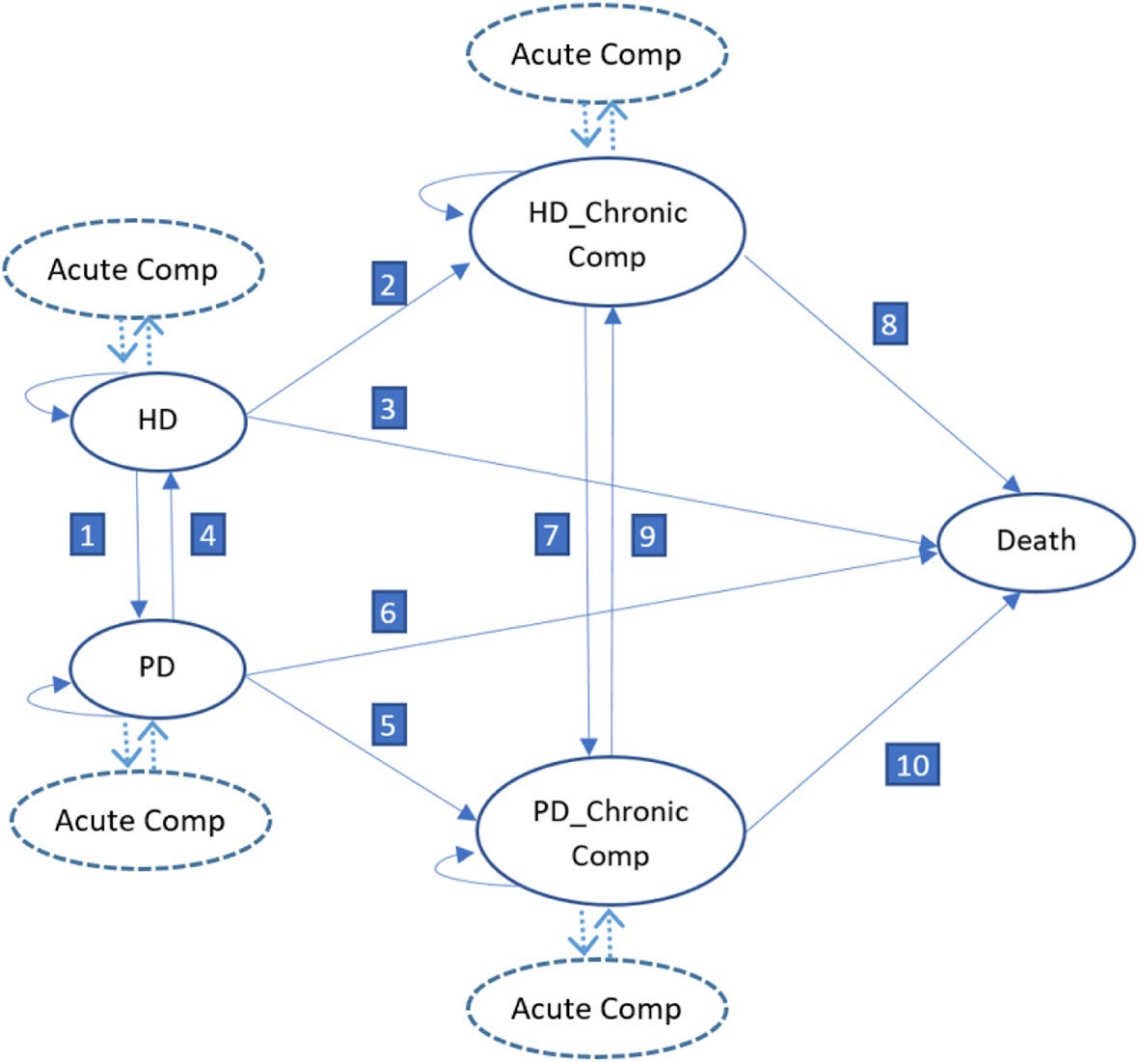
A structure of Markov model for dialysis modalities. Acute Comp = acute complications, Chronic Comp = chronic complications, HD = hemodialysis, PD = peritoneal dialysis

A 5-state Markov model was constructed, see Fig. 1. ESKD patients entered the model with HD or PD state (PD referred to solely as CAPD) depending on which strategy they first received. For each Markov cycle of one year, patients may stay in the same state or transition to other states, including modality switching (PD◊HD, HD◊PD) or with chronic complications (i.e., cardiovascular disease, cerebrovascular disease/stroke, chronic kidney disease-related mineral, and bone disorders given no reverse transition). In addition, for each cycle, patients may temporarily have acute complications (i.e., vascular access infection or peritonitis) which can be cured. The model was run until all patients qualified for the absorbing state as death with an annual discount rate of 3%.

### Model parameters

Table 1 summarised the parameters used in the model. Information about data retrievals and collections are as follow:

**Table 1:** Markov model parameters

Parameter	Start with PD	Start with HD	Distribution	Source
Annual transition probabilities				
Change mode (PD to HD or HD to PD)	0.039 (0.035, 0.044)	0.0073 (0.0065, 0.0081)	Beta	Electronic data from 15 hospitals and TRT
PD to PD with chronic complications	0.162 (0.148, 0.177)	0.557 (0.445, 0.698)	Beta	
HD to HD with chronic complications	0.301 (0.248, 0.366)	0.168 (0.162, 0.175)	Beta	
Acute complications				
- PD	0.052 (0.050, 0.054)	0.036 (0.035, 0.036)	Beta	
- PD with chronic complications	0.106 (0.103, 0.111)	0.080 (0.079, 0.082)	Beta	
- HD	0.154 (0.146, 0.163)	0.256 (0.243, 0.270)	Beta	
- HD with chronic complications	0.203 (0.164, 0.250)	0.138 (0.113, 0.168)	Beta	
- Death rate	Survival regression (Weibull distribution)			
Costs				
* Direct medical cost*				Electronic data from 15 hospitals, TRT, and expert opinion
Set up cost	208.52 (± 10%)	357.46 (± 10%)		
Annual cost				
- No chronic complications	7,955.20 (7,031.28, 9,079.12)	9,079.12 (7,915.28, 10,943.22)	Exponential	
- With chronic complications	9,220.85 (7,56145, 11,967.08)	12,120.91 (8,800.51, 19,493.12)	Exponential	
- With acute complications (add-on)	251.06 (27.70, 685.43)		Exponential	
- With both acute and chronic complications (add-on)	487.55 (97.68, 1,104.71)		Exponential	
* Direct non-medical cost*				Survey from 11 hospitals
No chronic complications	98.90 (44.53, 152.22)	1,519.58 (586.15, 2,101.44)	Gamma	
With chronic complications	215.22 (96.89, 331.55)	1,546.07 (596.34, 2,138.00)	Gamma	
* Indirect cost*				
No chronic complications	78.46 (0, 143.77)	1,004.71 (0, 3,157.69)	Gamma	
With chronic complications	170.75 (0, 313.15)	1,022.22 (0, 3,212.64)	Gamma	
Utility scores				
No chronic complications	0.731 (0.689, 0.772)	0.811 (0.786, 0.837)	Beta	Survey from 11 hospitals
With chronic complications	0.599 (0.5060.692)	0.639 (0.567, 0.712)	Beta	

*HD *Hemodialysis, *PD *Peritoneal dialysis, *TRT *Thai Renal Replacement Therapy

- The transition probability of change state was estimated using clinical databases from 15 hospitals plus HD-registry under supervision of the Thai Renal Replacement Therapy Association.
- The cost consisted of direct medical costs that were retrieved from electronic databases of the same sources above plus expert opinion, whereas direct non-medical and indirect costs were prospectively collected by using a cross-sectional survey from 11 hospitals across the country from January to May 2020.
- The utility data using EuroQoL-5D was collected from a cross-sectional survey of 11 hospitals.

#### Transition probability

The transitional probabilities for all states were estimated using the clinical real-practice data retrieved from the 15 participating hospitals, which consisted of 10,252 adult ESKD patients who underwent initial dialysis either PD (*N* = 1,826) or HD (*N* = 8,426) from 2008 to 2018. The transitional probabilities of death in the cycle were estimated using a Weibull survival regression by regressing survival time on covariables including age, gender, reimbursement, region, type of hospital, diabetes, hypertension, and chronic complications (i.e., cardiovascular disease and chronic kidney disease-related mineral and bone disorders). Survival rates were estimated by age at starting dialysis and adjusted by other covariables. The transitional probabilities of changing health states (i.e., the probability of switching modality and the probability of acute/ chronic complication occurrence) were estimated using the Kaplan–Meier approach to inform the yearly transition probabilities in the model.

#### Measurement of costs

The direct medical cost was calculated from 15 financial databases by the following six steps plus additional cost from the Thai Renal Replacement Therapy Association dataset and expert opinion as follows: (1) the medical cost types were re-categorised into eight groups out of 189, i.e., lab, medical supply, medicine, operation, room, service, X-ray, and other costs; then a total cost was aggregated accordingly. (2) each patient visit was aggregated within 30 days as one observation by summation of all cost groups. (3) the cost data from 15 hospitals were then merged into one large study cohort. (4) the costs were adjusted to the year 2021 according to the inflation rate from the National Bank of Thailand. (5) clinical outcomes were categorised according to complications, including without complication, chronic complication, acute complication, and chronic with acute complication. Therefore, there were eight groups of patients in total. (6) descriptive statistics for the total cost accounted adjusted for inflation rate were estimated by treatment groups. Direct medical costs that were excluded in hospital billing data were considered using the data from the Thai Renal Replacement Therapy Association and expert opinion, including set up cost, erythropoietin cost, HD cost from outside study hospitals, and PD solutions cost. All costs were adjusted to 2021 and converted to USD (1 USD = 33.57 Thai Baht) [23].

Direct non-medical costs included traveling, extra food/accommodation, and caregiver costs. Indirect costs represent a productivity loss in both patient and caregiver due to undergoing dialysis, i.e., income lost from taking sick leave. The unit costs per visit of direct non-medical, and indirect costs were calculated using median and interquartiles. Then, the total of both costs was also calculated by multiplying the unit cost per visit with the number of follow-ups or hospitalisation days in case of admissions.

#### Valuation of utility

The EuroQoL-5D was a self-reported questionnaire consisting of five dimensions (i.e., mobility, self-care (washing or dressing myself), usual activities (such as work, study, housework, family or leisure activities, pain/discomfort, and anxiety/depression). Each question was graded based on severity as no problems to unable/extreme problems, then converted into a utility score based on the Thai population [24]. The score of patients with and without complications (i.e., infection, cardiovascular disease, cerebrovascular disease/stroke, and chronic kidney disease-related mineral and bone disorders) were then estimated by PD and HD groups using inverse probability weighting with regression adjustment (IPWRA) as follows: the probability of treatment assignment (PD or HD) was estimated using a logit model by fitting treatment variables (i.e., PD and HD) on covariables: which included age, education level, income level, region, reimbursement, fracture, diabetes, hypertension, dyslipidaemia, and cardiovascular disease. Then, the outcome model (i.e., utility score) was constructed using a linear regression weighted by inverse probability and adjusted for some covariables, including age, gender, income level, region, fracture, and complications. The potential outcome means of the utility score by treatments and complications were then estimated. Quality adjusted life-year (QALY) was then calculated by multiplying the utility score with life-years gained as the outcome measurement.

### Deterministic and one-way sensitivity analysis

Deterministic analysis was conducted for five age groups (i.e., 20, 30, 40, 50, and 60 years) based on societal and government perspectives; the former considered direct medical- and non-medical costs and indirect costs, whereas the latter considered only direct-medical costs. Lifetime cost, life years, and QALY were then estimated for each treatment and scenario, and then an incremental cost-effectiveness ratio (ICER) was calculated by dividing the difference in lifetime cost by the difference of QALY between PD and HD arms. Lifetime cost included direct medical, direct non-medical, and indirect costs from the societal perspective, whereas only direct medical cost was accounted for from the government perspective. One-way sensitivity analysis was performed for mean dialysis at an initiated median age of 55.7 years to test the robustness of the model by varying each parameter individually to the high or low value for illustrating the influential parameters affecting the ICER.

### Probabilistic sensitivity analysis and cost-effectiveness acceptability curve

Probabilistic sensitivity analysis was applied by running 5,000 iterations to examine how parameters affected the ICER. Gamma and exponential distributions were used for age and cost, whereas beta distribution was used for the rest. The results are presented as an ICER plane and cost-effectiveness acceptability curve.

## Results

### Deterministic analysis

The results of five age groups at 20, 30, 40, 50, and 60 years in both societal and government perspectives are presented in Table 2. The lifetime costs for PD-first (range: 39,802 to 96,355 USD in societal and 37,837 to 90,204 USD in government perspective) were lower than those with HD-first (range from 73,311 to 166,600 USD in societal and 58,243 to 132,084 USD in government perspective) in all scenarios. For health outcomes, HD-first patients were expected to gain 1.17 to 1.99 LYs and 1.05 to 1.76 QALYs over patients with PD-first. When compared to the PD-first strategy, although HD-first had better outcomes in all aspects, the lifetime cost was more expensive with the ICER of 31,913 to 39,912 USD per QALY in societal and 19,434 to 23,796 USD per QALY in government perspective.

**Table 2:** Incremental cost-effectiveness ratio among PD vs. HD first strategies by age group in government and societal perspectives

**Age**	**LY** **(years)**		**QALY** **(years)**		**Life time cost** **(USD)**		**Cost per LY** **(USD per year)**		**ICER** **(USD per QALY)**
	PD	HD	PD	HD	PD	HD			
Government perspective									
20	9.85	11.84	6.43	8.19	90,204	132,084	9,158	11,156	23,796
30	8.42	10.37	5.53	7.22	76,613	114,911	9,099	11,081	22,662
40	6.71	8.36	4.46	5.91	60,255	91,366	8,980	10,929	21,456
50	5.40	6.80	3.63	4.87	47,873	73,243	8,865	10,771	20,460
60	4.32	5.49	2.94	3.99	37,837	58,243	8,759	10,609	19,434
Societal perspective									
20	9.85	11.84	6.43	8.19	96,355	166,600	9,782	14,071	39,912
30	8.42	10.37	5.53	7.22	81,713	144,894	9,705	13,972	37,385
40	6.71	8.36	4.46	5.91	63,993	115,143	9,537	13,773	35,276
50	5.40	6.80	3.63	4.87	50,613	92,251	9,373	13,566	33,579
60	4.32	5.49	2.94	3.99	39,802	73,311	9,213	13,353	31,913

*HD* Hemodialysis, *ICER *Incremental cost-effectiveness ratio, *LY *Life year, *PD *Peritoneal dialysis, *QALY *Quality adjusted life year

#### One-way sensitivity analysis

**Fig. 2:**
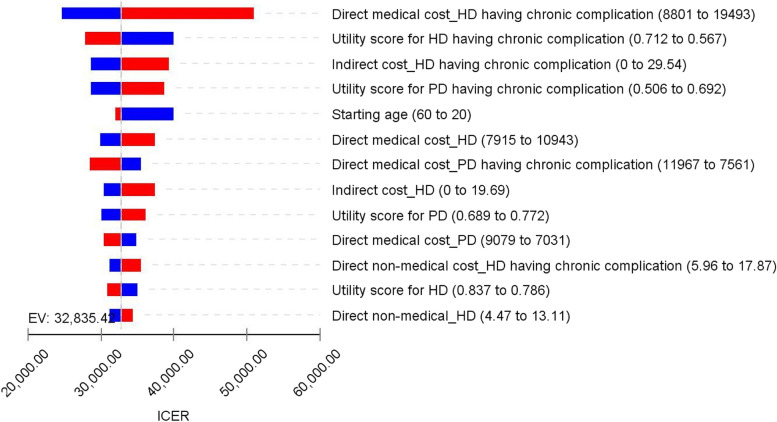
Tornado diagram of incremental cost-effectiveness ratio (ICER) between hemodialysis (HD) vs. peritoneal dialysis (PD). The blue bar section represents the parameter range from the low uncertainty value to the base case, while the red bar section represents the parameter range from the base case to the high uncertainty

The results of the one-way sensitivity analysis are presented in Fig. 2. At the mean age of 55.7 years from the societal perspective, the ICER was most sensitive to the change in the direct medical cost of HD with chronic complications, followed by the utility score of HD with chronic complications and indirect cost of chronic complications, respectively. ICER varied from 22,000 to 51,000 USD per QALY.

### Probabilistic sensitivity analysis

**Fig. 3:**
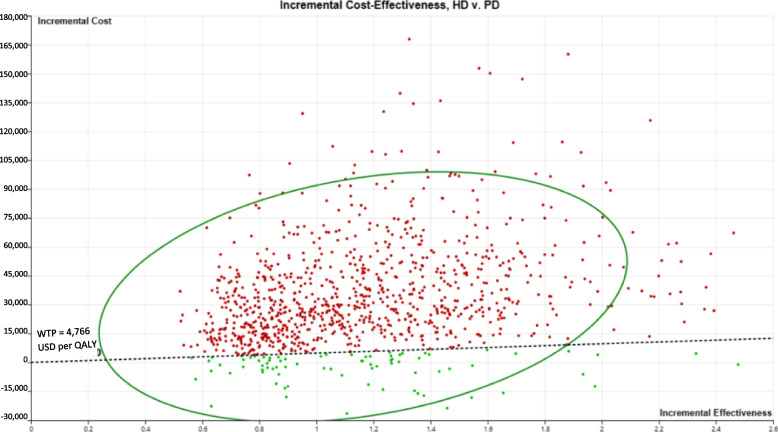
Incremental cost-effectiveness ratio plane by a Monte Carlo simulation in societal perspective

After 5,000 iterations, the average lifetime cost of HD-first and PD-first were 91,458 and 57,282 USD with a mean difference of 34,176 USD, respectively. The average QALYs of HD and PD-first were 4.28 and 3.19 QALYs along with a mean difference of 1.10 QALYs. The results of probabilistic sensitivity analysis are illustrated in Fig. 3, which were consistent with those of the deterministic analysis results.

**Fig. 4:**
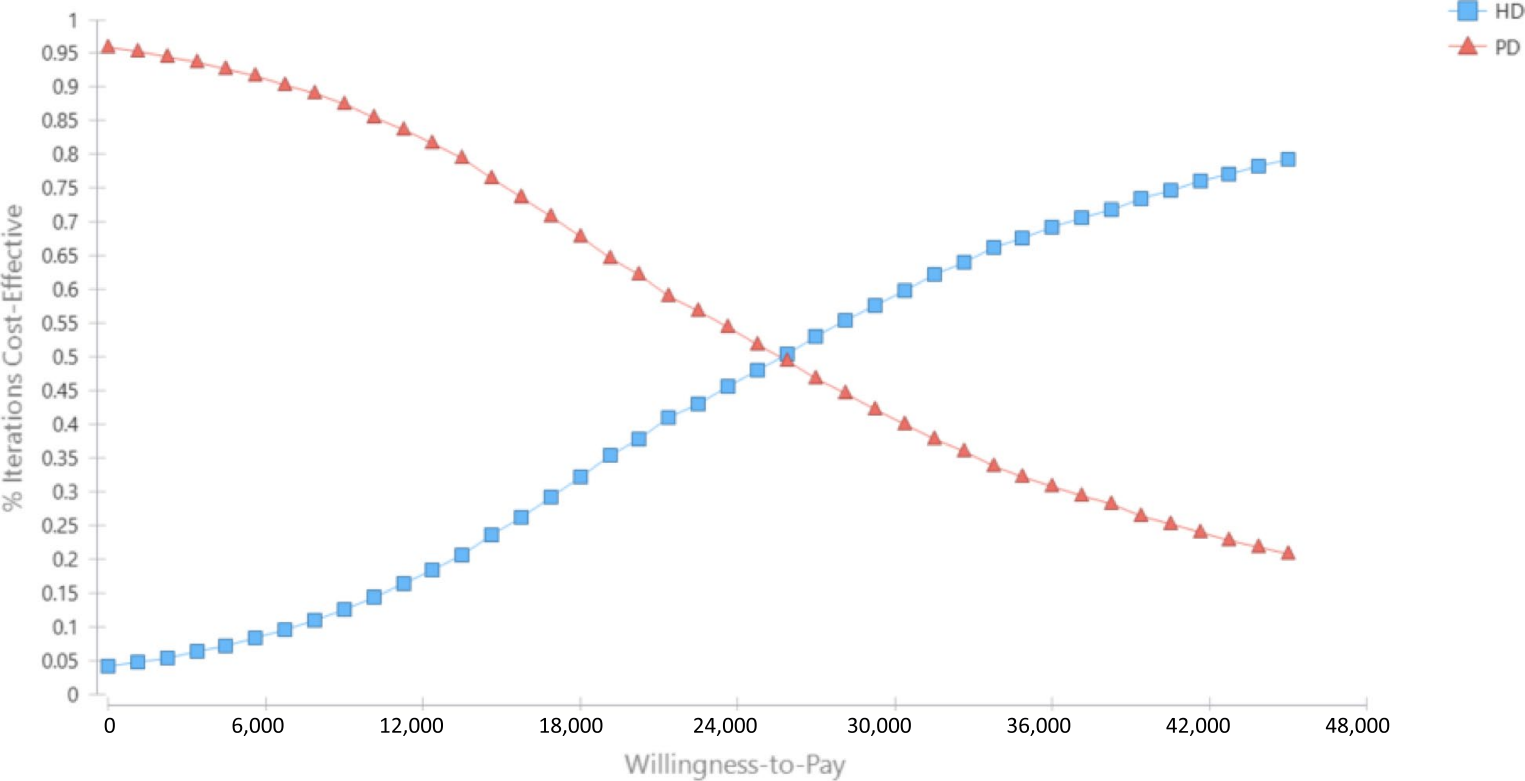
Cost-effectiveness acceptability curve of hemodialysis (HD) vs. peritoneal dialysis (PD)

The cost-effectiveness acceptability was constructed by plotting the percent coverage of cost-effectiveness on Y axis and willingness-to-pay on X axis by PD and HD modalities, see Fig. 4. This suggested that the PD-first was currently still more cost-effective than the HD-first strategy given the current willingness to pay in Thailand of 4,766 USD per QALY. The strategy will shift, i.e., HD is more cost-effective than PD when the willingness to pay rises to 25,320 USD per QALY.

## Discussion

We conducted the study using real-practice data across the country to perform a cost-utility analysis of dialysis therapies for ESKD patients in Thailand. Comparing HD with PD in age groups from 20 to 60 years indicated that the ICERs were 19,434 to 24,796 USDs and 31,913 to 39,192 USDs per QALY gained in governmental and societal perspectives, respectively. As a result, the HD was not cost-effective as an initial treatment relative to the PD from both governmental and societal perspectives given the current willingness to pay threshold in Thailand of 4,766 USD per QALY. The results were also validated using a probabilistic sensitivity analysis, and were consistent with the deterministic results. One-way sensitivity analysis revealed that the direct medical cost of HD with chronic complications was the most sensitive parameter for the cost-effectiveness of PD versus HD. These findings have substantial implications regarding optimising the quality of ESKD care and preventing chronic complications, which would have a paramount impact on cost-effectiveness outcomes.

Our findings were similar to previous cost-effectiveness analyses, which found that PD was more cost-effective than HD [10, 25–29] including the study conducted in Thailand in 2007; although this study found that both PD and HD were not cost-effective when compared with palliative care, while PD was cost-effective relative to HD. In addition, a few studies conducted in South East Asia also found consistent results that PD was more cost-effective than HD from both government [30] and societal perspectives [25, 28]. However, a few studies reported contrary results, which indicated HD was almost as cost-effective as PD [31] or more cost-effective than PD [32]. Those inconsistent findings may be explained by the differences in country context and the source of input parameters used. For decades, the effectiveness of dialysis modalities in terms of patients’ survival has been debated due to the difficulty of conducting randomised control trials [15, 33]. The sources of input parameters regarding patient survival were also varied in the economic evaluation, which may have impacted the outcome. Some cost-effectiveness analyses used survival input parameters from a single dialysis modality and assumed that HD and PD had similar survival outcomes [10, 25], while others analysed data from national registry databases and used distinct input parameters [28, 29, 31]. A prior economic evaluation of kidney replacement therapy in Thailand that compared HD and PD to palliative care assumed that PD and HD had equivalent survival outcomes. The results revealed that the PD strategy dominated the HD strategy when PD survival was assumed to be equal to HD survival, which was obtained from the Thai Renal Replacement Therapy Registry^7^. Our study used transitional probabilities derived from real-practice data of 10,252 adult ESKD patients who initiated dialysis with PD or HD between 2008 and 2018 in 15 hospitals located throughout Thailand. We also used a Weibull regression with the adjustment of covariables (i.e., age, gender, reimbursement, region, type of hospital, diabetes, hypertension, and chronic complications related to chronic kidney disease (i.e., cardiovascular disease and chronic kidney disease-related mineral and bone disorders)) [34] that could affect patients’ survival indicating HD gained more life years and quality-adjusted life years than PD. We were concerned that the dialysis modality selection was not randomised due to reimbursement policy and doctor/patient preferences, which could result in selection bias and invalid findings. Therefore, we estimated treatment effects using the propensity score method with inverse probability weighting regression adjustment to minimise selection bias. Interestingly, despite real-practice data in our country showing that HD results in a higher QALY gain than PD due to better patient survival and utility, as shown in Table 2, PD has remained more cost-effective than HD due to PD’s significantly lower lifetime treatment cost.

Our analysis demonstrated that the cost-effectiveness acceptability curve would shift to HD is more cost-effective than PD when willingness to pay of Thailand reaches 25,320 USD per QALY, i.e., approximately five times higher than the current Thailand’s willingness to pay and 3.5 times Thailand’s GDP per capita in 2021 at 7,186 USD. Those findings provide information on the cost-utility of ESKD treatments in Thailand, while the decision depends upon the healthcare system’s willingness to absorb incremental costs associated with HD.

The strength of our study was the newly developed Markov model, which included acute complications such as vascular access infection/dysfunction or peritonitis, as well as chronic complications such as cardiovascular disease, cerebrovascular disease, and chronic kidney disease-related mineral and bone disorders in ESKD patients [34]. This model more accurately represented the natural history of diseases than previous studies, which only included dialysis mode transition and death. More updated data across the country were used, including cost, disease progression (i.e., CKD-related chronic complications and mortality) from 15 hospitals, utility data from a cross-sectional survey of 11 hospitals plus the data from Thai Renal Replacement Therapy Registry. Although these data were only in some parts of the whole country, they mostly reflect the current practices and setting of Thailand. Third, the analyses were conducted based on age at dialysis initiation, which is a significant component in the cost-utility analysis outcomes.

Nonetheless, our study had several limitations due to the following factors: first, we did not consider other modalities (e.g., kidney transplantation, automated peritoneal dialysis, and hemodiafiltration) that are currently applied for kidney replacement therapy in ESKD patients in Thailand. However, KT rate was only 3.6% [35] and automated PD accounts for approximately 3% of peritoneal dialysis in Thailand [36]. We also needed to estimate some parameters not included in hospital billing, such as erythropoiesis-stimulating agents and PD solution from Thailand renal replacement therapy registry, in addition to individual-level cost data from hospital-electronic records due to the complexity of ESKD management’s cost structure and the Thai reimbursement system. However, we conducted a one-way sensitivity analysis on all parameters to ensure the robustness of our results.

## Conclusion

On average, HD-first is not cost-effective when compared to PD-first given the willingness to pay threshold of 4,766 USD. However, it is essential to note that the QALY gained from HD is higher than the PD based on current Thailand’s real-practice data, thus individualised patients may gain benefit from HD. Hence, this information will assist clinicians and policymakers in determining the future direction of dialysis modality selection and kidney replacement therapy reimbursement policies for ESKD patients.

## Supplementary Information

**Additional file 1: Appendix A.** Hospital’s list.

## Data Availability

The authors confirm that the data supporting the findings of this study are available within the article and its supplementary materials.

## Abbreviations

CAPD: Continuous ambulatory peritoneal dialysis
CUA: Cost-utility analysis
ESKD: End-stage Kidney Disease
EuroQoL-5D: European quality of life – 5 dimensions
HD: Hemodialysis
ICER: Incremental cost-effectiveness ratio
IPWRA: Inverse probability weighting with regression adjustment
IRB: Institutional review board
KT: Kidney transplantation
LY: Life-year
PD: Peritoneal Dialysis
QALY: Quality adjusted life-year
USD: United States dollar

## Declarations

All the methods were carried out in accordance with relevant guidelines and regulations, and informed consent was obtained from all subjects.
