# Supplementary material for: Economic evaluation of peritoneal dialysis and hemodialysis in Thai population with End-stage Kidney Disease
Source: BMC Health Serv Res. 2022 Nov 21;22:1384. doi: 10.1186/s12913-022-08827-0 (PMC9677653; doi:10.1186/s12913-022-08827-0)
Supplement: Supplementary file 1 — Additional file 1: Appendix A. Hospital’s list. [file 12913_2022_8827_MOESM1_ESM.docx]

Appendix A: Hospital’s list

| **Hospital’s list** | |
| --- | --- |
| 1. **15 hospitals provided clinical and financial databases** | 1. **11 hospitals participated with a cross-sectional survey for**   direct-non medical, indirect costs, and utility data |
| 1.Ramathibodi Hospital | 1.Ramathibodi Hospital |
| 2.Chiangmai Hospital | 2. Bhumibol Adulyadej Hospital |
| 3.Bhumibol Adulyadej Hospital | 3. Siriraj Hospital |
| 4. Khon Kaen Hospital | 4. King Chulalongkorn Memorial Hospital |
| 5. Suratthani Hospital | 5. Suratthani Hospital |
| 6.Thammasat Hospital | 6. Hatyai Hospital |
| 7. Buddhachinaraj Hospital | 7. Panyananthaphikkhu Chonprathan Hospital |
| 8. Ratchaburi Hospital | 8. Ratchaburi Hospital |
| 9.Nakhonpranom Hospital | 9. Buddhachinaraj Hospital |
| 10. Maesot Hospital | 10. Maesot Hospital |
| 11. Hatyai Hospital | 11. Khon Kaen Hospital |
| 12.UthaiThani Hospital |  |
| 13.Taksin Hospital |  |
| 14. Panyananthaphikkhu Chonprathan Hospital |  |
| 15.Surin Hospital |  |
